# Stepwise occlusion of the carotid arteries of the rat: MRI assessment of the effect of donepezil and hypoperfusion-induced brain atrophy and white matter microstructural changes

**DOI:** 10.1371/journal.pone.0198265

**Published:** 2018-05-31

**Authors:** Gabriella Nyitrai, Tamás Spisák, Zsófia Spisák, Dávid Gajári, Pálma Diószegi, Tamás Zsigmond Kincses, András Czurkó

**Affiliations:** 1 Preclinical Imaging Center, Pharmacology and Drug Safety Research, Gedeon Richter Plc., Budapest, Hungary; 2 Department of Neurology, Albert Szent-Györgyi Clinical Center, University of Szeged, Szeged, Hungary; Hungarian Academy of Sciences, HUNGARY

## Abstract

Bilateral common carotid artery occlusion (BCCAo) in the rat is a widely used animal model of vascular dementia and a valuable tool for preclinical pharmacological drug testing, although the varying degrees of acute focal ischemic lesions it induces could interfere with its translational value. Recently, a modification to the BCCAo model, the stepwise occlusion of the two carotid arteries, has been introduced. To acquire objective translatable measures, we used longitudinal multimodal magnetic resonance imaging (MRI) to assess the effects of semi-chronic (8 days) donepezil treatment in this model, with half of the Wistar rats receiving the treatment one week after the stepwise BCCAo. With an ultrahigh field MRI, we measured high-resolution anatomy, diffusion tensor imaging, cerebral blood flow measurements and functional MRI in response to whisker stimulation, to evaluate both the structural and functional effects of the donepezil treatment and stepwise BCCAo up to 5 weeks post-occlusion. While no large ischemic lesions were detected, atrophy in the striatum and in the neocortex, along with widespread white matter microstructural changes, were found. Donepezil ameliorated the transient drop in the somatosensory BOLD response in distant cortical areas, as detected 2 weeks after the occlusion but the drug had no effect on the long term structural changes. Our results demonstrate a measurable functional MRI effect of the donepezil treatment and the importance of diffusion MRI and voxel based morphometry (VBM) analysis in the translational evaluation of the rat BCCAo model.

## Introduction

An increasing amount of evidence has supported the notion that chronic cerebral hypoperfusion is deeply involved in several forms of cognitive impairments, including Alzheimer’s disease (AD) [1–5]. The reduction of blood flow and the concomitant pathological processes considerably impair the cerebral white matter and lead to gray matter atrophy revealed by neuroimaging [2, 6–8]. Consistently, these abnormalities have been shown to be related to cognitive impairment [1, 2, 9] and are also involved in the pathology of AD [10].

Permanent bilateral occlusion of the common carotid arteries (BCCAo) in rats can mimic several pathological features of human cerebral hypoperfusion, such as myelin degeneration and astrogliosis in the white matter and long-lasting cognitive impairment [11–15]; therefore, the BCCAo model is often used in pharmacological studies that evaluate the effects of procognitive compounds [16–19].

Cholinergic dysfunction has been reported after BCCAo [11, 20–22], and memory impairments due to brain hypoperfusion were successfully improved by donepezil [16–18], an acetylcholinesterase inhibitor used in the symptomatic treatment of AD and vascular cognitive impairment [23, 24]. Magnetic resonance imaging (MRI) commonly used to monitor human dementia, was demonstrated to be a perfect translatable tool to follow white matter changes [25] focal ischemic lesions [26] or brain perfusion and arteriogenesis [26–28] after the classical BCCAo in the rat brain. Importantly, the sudden and relatively serious decrease in cerebral blood flow [11, 28], as the acute effect of the simultaneous occlusion of both carotid artery resulted in variable and high mortality rate together with a frequent occurrence of serious ischemic lesions [11, 19, 26]. Therefore, a recently introduced modification of the BCCAo model, the stepwise occlusion of the two carotid arteries, is of particular importance to separate the acute and chronic effects. While such an intervention was shown to induce cognitive deficit, the primary reason to introduce the stepwise occlusion was to reduce the mortality rate [29–33].

In this study, we used ultrahigh field multimodal MRI to assess the effects of semi-chronic (8 days) donepezil treatment in this model and to longitudinally monitor the effects of the chronic hypoperfusion up to 5 weeks after the stepwise BCCAo by objective translatable MRI measures. To disclose potentially valuable translational measures, we monitored the integrity of the white matter microstructure by diffusion tensor imaging (DTI) and the macrostructural alterations by voxel-based morphometry (VBM). We also examined the functional integration in response to whisker stimulation via blood oxygen level dependent (BOLD) functional MRI after the stepwise BCCAo, and brain perfusion with dynamic susceptibility-weighted contrast-enhanced (DSC)-MRI perfusion imaging.

## Materials and methods

### Animals and procedure

Thirty male Harlan Wistar rats (3 months old, weighing 314–370 g at the beginning of the experiments) were used. The animals were kept in polycarbonate cages in groups of 5 number of cage companions, bedding material, paper tubes and wooden blocks were put in for environmental enrichment in a thermostatically controlled room at 21±1°C. The room was artificially illuminated from 6 a.m. to 6 p.m. The rats were fed conventional laboratory rat food (sniff R/M + H Spezieldiäten GmbH D-59494 Soest). The acoustic noise of the gradient coil was driven through a plastic pipe system into the animal room to habituate the rats to the scanner noise. All of the procedures conformed to the National Institutes of Health’s guidelines for the care and use of laboratory animals and were approved by the local ethical committee (Scientific and Research Ethics Committee of the Medical Research Council, Ministry of Health, Medical Research Council, Budapest, Hungary). The experiments were performed and reported according to the ARRIVE guidelines on animal research.

The rats were anesthetized before transferring them to the magnet room. The anesthesia was introduced with 5% isoflurane and then maintained at 1–2% during scanning. During the experiment, the body temperature of each rat was maintained at 37 ± 1°C with thermostatically controlled air flow around each rat. The respiration of the animal was monitored continuously during the experiment with a small pneumatic pillow sensor (SA Instruments, Inc., NY, USA).

### Stepwise occlusion of the common carotid arteries

The rats were anesthetized with isoflurane (1.5–2% in air), and a ventral midline incision was made on the neck. The left common carotid artery was exposed, gently separated from the vagus nerve and occluded in this first step by three ligatures (2–0). One week later, a new incision was made, and the right carotid artery was occluded similarly. The wounds were then closed. After the procedures, the rats were put back in their home cages and allowed to recover for 1 week. Animals were treated with Baytril (5 mg/kg, s.c.,) and Norocarp (5 mg/kg, s.c.) post-operatively, and at least 7 days were allowed for recovery from the surgery.

### Experimental design

The rats were divided into 2 groups, n = 15 each. Before the occlusion, baseline functional MRI images and detailed morphological MRI scans were taken. After the occlusion of both the carotid arteries, one group received donepezil (3 mg/kg/day i.p.) from the 7^th^ day to the 14^th^ day (counted from the second occlusion), while the other group (control group) received i.p. injections of physiological saline daily (2 ml/kg/day). Animals were allocated into the treatment groups randomly, marked with increasing numbers (1–30) and received donepezil and saline treatment alternately. Because the duration of the MRI measurements were 1–1.5 hour, carotid occlusion and treatments were shifted in time as only a limited number of animals could be measured per day.

**Fig 1:**
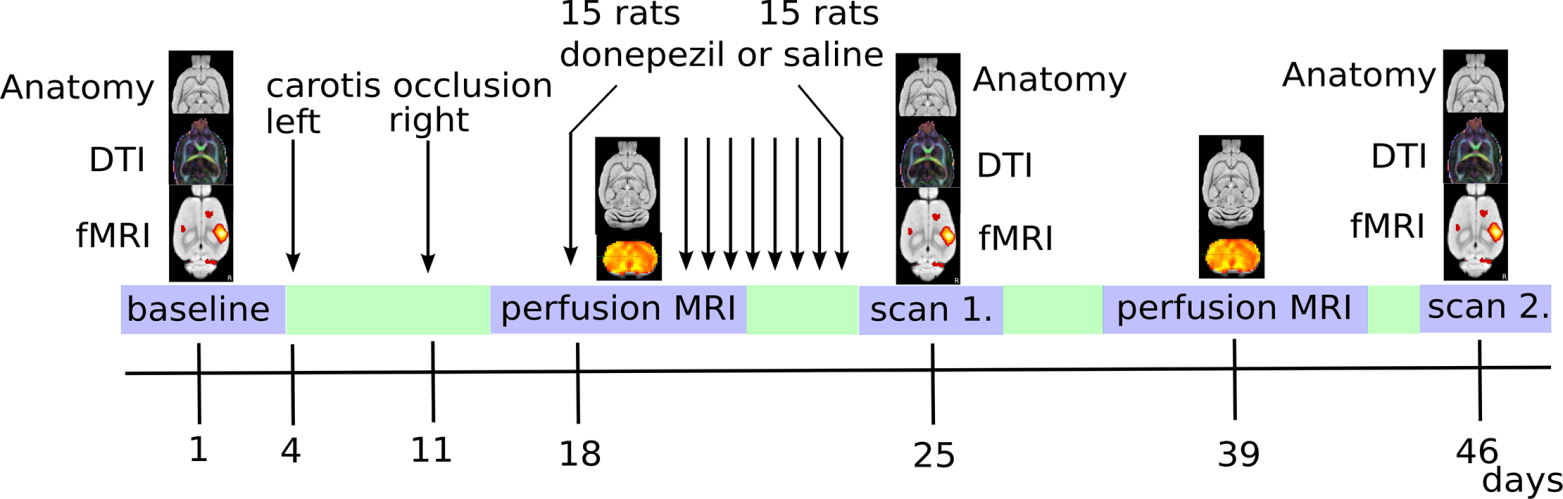
Experimental design. After the baseline scans (PD: structural, fMRI: whisker BOLD, DTI: diffusion MRI, n = 30), the carotid vessels were ligated on each side one week apart. After a week of recovery, a semi-chronic (8 days) donepezil (3 mg/kg i.p., n = 14) or saline (n = 14) treatment was initiated. The first perfusion and structural (PD) measurements were carried out after the first dose of drug (donepezil or saline) and one week after the second carotid ligation. After the end of the semi-chronic treatment regime, functional, detailed structural and diffusion scans were acquired (scan 1). Two weeks later, second perfusion and structural scans were acquired, and then an additional week later, functional, detailed structural and diffusion scans were acquired (scan 2).

The first post-intervention DSC-MRI perfusion measurements were carried out just after the first dose of the semi-chronic drug treatment (donepezil or saline), while the second DSC-MRI perfusion was carried out 2 weeks after the end of the semi-chronic drug treatments. The second functional MRI images were taken just after the semi-chronic drug treatments (2 weeks after the 2nd occlusion), and the third functional MRI scans with detailed morphological MRI were performed 5 weeks after the occlusion of the second carotid artery (Fig 1) Note, that the planned measurements required long-lasting MRI scans, and to minimize the side effects of the repeated long-term anesthesia we needed to optimize our measurements.

### Image acquisition

The MRI experiments were performed using a 9.4 T Varian MRI system (Varian Associates Inc., Palo Alto, CA) with a free bore size of 210 mm, which contained a 120 mm inner size gradient coil (minimum rise time 140 μs, 200 μs were used). For excitation, an actively RF-decoupled 2 channel volume coil system with inner size of 90 mm was used and a fix tuned receive-only phase array rat brain coil (RAPID Biomedical GmbH, Rimpar, Germany) was located directly above the dorsal surface of each rat’s head to maximize the signal-to-noise ratio.

Scout images were taken in coronal and sagittal planes in order to set the voxel-based shimming region for the anatomical and functional images.

Before the perfusion measurements (Fig 1, 1 and 4 weeks after BCCAo), we acquired short, proton density-weighted anatomical scans using a gradient echo multi slice (echo time: 3.83 ms, repetition time: 200 ms, flip angle: 45°, averages: 1, dummy scans: 4, data matrix: 256×256, total scan time: 45 sec) sequence with a field of view of 40 mm×40 mm and a slice thickness of 0.6 mm without an inter-slice gap (resolution: 0.156×0.156×0.6 mm^3^). Twenty-four slices were acquired in an interleaved order.

In the baseline measurement, for scan 1 and scan 2 (Fig 1; before, 2 and 5 weeks after BCCAo) we acquired proton density-weighted anatomical scans using a gradient echo multi slice (GEMS: echo time (TE) = 5.78 ms, repetition time (TR) = 855 ms, flip angle = 40°, data matrix: 256×256, total scan time: 22 min 30 sec) sequence (FOV = 40 mm×40 mm, slice thickness 0.2 mm, without inter-slice gap). Seventy-four slices were acquired in an interleaved order. Anatomical scans were repeated 8 times and then averaged into a single image.

Diffusion-weighted images were acquired with an echo planar imaging (EPI) sequence with a spin echo train, (TE = 36.24 ms, TR = 2180 ms, 60 directions, b-value = 1002.2 s/mm^2^, 40 slices, FOV = 40×40 mm, matrix size = 134×134, total acquisition time = 40 min) with a spatial resolution of 0.3×0.3×0.3 mm.

### Dynamic susceptibility-weighted contrast-enhanced (DSC–MRI) perfusion imaging

An echo planar imaging (EPI) sequence was used for cerebral DSC-MRI: TE = 5 ms, TR = 1000 ms, flip angle 90°, averages: 1, dummy scans: 4, number of slices = 16, FOV = 40 x 40 mm, matrix size = 50 x 50 pixels, resulting in a spatial resolution of 0.8 x 0.8 mm in 0.8 mm slice thickness, and 850 repetitions were recorded consecutively within a total acquisition time of 14 min 10 s. Three shots with compressed segments, ramp sampling and a single phase correction reference scan were used to acquire the EPI images, and the recording time per volume was 1 s. The first 4 min (240 volumes) were used as a baseline, then a bolus of 0.3 ml (1 mmol/ml) of gadobutrol (Gadovist, Bayer Pharma AG D-13342 Berlin) was administered via the tail vein over an 8.06 s interval (8 volumes) by a remotely controlled perfusor at a rate of 134 ml/h for each animal. The hypodermic needle in the tail had been filled with 0.1 ml of saline, and an effective dose of 0.2 ml contrast agent /animal was used as a bolus. The experiment finished with an additional 10 min and 2 s of recording (602 volumes).

### Functional MRI experiments

The T2*-weighted EPI sequence for the functional MRI experiments had the following modifications compared to the similar setting used in DSC-MRI: TE = 10 ms, TR = 2030 ms, flip angle: 90°, averages: 1, dummy scans: 4, data matrix: 50 × 50, 400 repetitions, FOV = 40 mm×40 mm, slice thickness: 0.8 mm, no inter-slice gap, and 16 horizontal slices (resolution: 0.8×0.8×0.8 mm^3^). A triple reference scan was used instead of a single; thus, the functional images were acquired with 2 opposite gradient polarities, which resulted in an effective TR of 4060 ms. Although this correction doubles the effective repetition time (4060 ms), it results in a significant reduction of the EPI Nyquist ghost artifact (See Varian vnmrJ Manual v2.2B and [34].

For a somatosensory stimulus, pneumatic whisker stimulation was used in a block-design fashion. The stimulation was delivered through a tubing system that was integrated into the holding cradle. The air pressure was set to 1 bar. Air puffs were delivered at a frequency of 1 Hz with a blowing time of 200 ms. The duration of the stimulation was 30 s followed by a 60 s rest. Over one experimental session, 9 blocks of stimulation were used. The pneumatic stimulation was controlled by a custom-programmed user interface developed in LabView (LabView, National Instruments, Austin, TX, USA).

### Data analysis

Throughout the image processing procedure, we intended to follow the conventional human practice for MRI analysis. Small-animal imaging-specific features of the applied image processing pipeline are described in detail.

### Preprocessing

The raw images were converted to NIfTI-format (Neuroimaging Informatics Technology Initiative) by an in-house developed script. The anatomical and functional images were reoriented in order to match the standard orientation of the digitalized version of the Rat Brain Atlas of Paxinos & Watson [35]. To preserve the directions of the B-vectors, DTI images were processed in the native space (without reorientation), and only the computed parametric maps were reoriented. All the images were rescaled by a factor of ten to achieve image dimensions similar to human data and thus facilitate the use of image processing algorithms developed for human image analysis [36, 37]. All the approaches performed on the up-scaled images were scale-invariant, except for a built-in constraint optimization for motion correction. The up-scaling only affects the dimension descriptor fields in the file header and does not involve interpolation or any information loss. Thus, it does not introduce any bias in statistical analysis, for example. The analysis was carried out in a multistage process using the image analysis software package, FSL [38] (FMRIB's Software Library, www.fmrib.ox.ac.uk/fsl), and in-house developed software tools. All the fMRI time-series were motion-corrected using MCFLIRT [39]. FSL BET (Brain Extraction Tool [40]) was used to remove non-brain areas from the structural and functional images. The fractional intensity threshold was set to 0.65 and 0.7 for functional and structural images, respectively, and the vertical gradient in the fractional threshold was set to 0.1. Before the brain extraction, the images were rescaled in the y-direction by a factor of 0.5 to ensure that the spherical brain model used by BET gained robust segmentation results. The brain-extracted images were then rescaled to the original y-axis dimensions.

To achieve spatial correspondence for the group analysis, all the images were spatially standardized using FSL FLIRT [39, 41], which utilized a 6-parameter rigid-body transformation. The high resolution structural image was fitted to an in-house developed standard template (average of 200 non-linearly fitted structural images) by an affine transformation (FLIRT) and a non-linear deformation field estimated by FNIRT [42]. In the latter co-registration procedure, a 10x10x10 mm^3^ warping field (in up-scaled space) was estimated with three iterations using a relatively conservative lambda parameter set of 60, 40 and 20. This stricter regularization was applied as the morphological diversity of the rat brain is smaller compared to humans.

### Analysis of perfusion (DSC-MRI) data

Dynamic susceptibility-weighted contrast-enhanced (DSC-MRI) perfusion imaging maps the temporal changes of T2*-intensity modulation caused by the contrast agent bolus and utilizes tracer kinetic theory to estimate various characteristics of cerebral perfusion [43].

Due to the differences between human and animal physiology and image acquisition, the preclinical usability of software tools developed for human DSC imaging is limited. Thus, we have developed an R-based (http://www.r-project.org) software package for DSC perfusion imaging of the rat brain. The main steps of the analysis are analogous to that of the human applications; however, several parameters of the process have been adapted and optimized to the special requirements of rat brain imaging.

As shown by [44], the gadolinium concentration is proportional to the change in the relaxation rate, ΔR2* (the reciprocal of T2*). It can be calculated from the signal by using the following equation:

$$\Delta {R2}^{*}=\ln\left(\frac{{SI}_{t}}{{SI}_{0}}\right)/TE$$

where SI_t_ is the signal intensity at time t, SI_0_ is the baseline intensity (calculated from the first 200 TRs before bolus administration) and TE is the echo time (assuming that the contrast agent does not pass the blood-brain barrier, which renders the T1 enhancement negligible).

In the absence of recirculation, cerebral blood volume (CBV) is proportional to the area under the ΔR2*. However, in general and especially in small animal imaging, this assumption is violated. Following the theory of tracer kinetics, the tissue-response alone can be modeled with a gamma-variate function [45], and the concentration of contrast agent originating from recirculated blood forms a sigmoid trend below the tissue response. Since the average recirculation time for blood is much shorter in rats than in humans, estimates of CBV and CBF might be biased. Therefore, modeling recirculation is of crucial importance. To achieve this, the measured ΔR2* can be modeled as a linear mixture of a sigmoid basis for recirculation and a gamma-variate function for tissue response.

This mixture model was fitted to the data in each voxel of the images with the nlsLM function of the minpack.lm R package [46], which utilizes the Levenberg-Marquardt fitting algorithm and thus, tolerates inaccurate starting values well. After fitting the data, the regional mean transit time (MTT) was estimated as the full-width at the half-maximum of the fitted gamma (considered to be proportional to the real mean transit time). The relative regional cerebral blood flow (rCBF) was calculated as the ratio of rCBV to MTT. Time to peak (TTP) was calculated as the difference between the index of the last frame of the baseline and the index of the frame with the maximal ΔR2* value. The first frame where ΔR2* reaches 10% of the maximal ΔR2* value within that voxel was considered as the first frame of the tissue response.

The perfusion parameter images were transformed into standard space using the transformation matrices and the deformation field estimated during standardization and co-registration. Perfusion differences between the saline and the donepezil groups were tested against zero with a voxel-wise, nonparametric permutation test implemented in the FSL randomize step [47]. The tests were performed with 10000 permutations for each contrast.

Perfusion parameters for whole brain were calculated as mean values from standardized images throughout the brain, and were compared using R lmperm package, permutation tests for linear models. Groups were represented as a factor variable, modelling the given perfusion parameter as dependent variable. Number of maximal permutations was set to 100000.

### Functional MRI (fMRI) analysis

Before the statistical analysis, the functional images were spatially smoothed using a Gaussian kernel of 12.0 mm FWHM (note that the images were up-scaled). High-pass temporal filtering (Gaussian-weighted least-squares straight line fitting, with sigma = 140.0 s) was then applied to remove slow drifts from the signal. The fMRI data processing was carried out using FEAT (FMRI Expert Analysis Tool) Version 6.00, which is part of FSL. After signal prewhitening (FILM), the time-series statistical analysis was carried out using the general linear model with local autocorrelation correction [48]. A first level analysis modeled each individual rat’s data from each session and included 7 explanatory variables in total: one regressor modeling the stimuli (convolved with a double-gamma canonical HRF), its temporal derivate in order to account for slight differences in timing, and 5 noise-ROI-based, CompCor [49] confounder variables. For each fMRI session, a noise ROI was delineated based on the temporal signal-to-noise ratio of the BOLD signals (the upper 2 percent of within-brain voxels were chosen on each slice), and the first five principal components of the corresponding time-series were extracted from the data following the widely used t-CompCor technique [49]. Individual statistical z-scored images for the first regressor were obtained (average BOLD response to the stimuli).

Using the spatial transformations computed during image co-registration and standardization, z-score maps resulting from the individual statistical analysis were realigned to the common standard space and based on them second-level statistical analysis was performed using FEAT [50]. First we compared baseline and measurements 2 and 5 weeks after BCCAo in the saline and in the donepezil group separately.

Measurements acquired two weeks and five weeks after the BCCAo were compared to the baseline measurements with a general linear model, implementing a repeated measures ANOVA design with one fixed and one mixed effect. The effect-of-interest (fixed factor) was the BCCAo (baseline, 2 and 5 weeks) and additional covariates modeled the individual subjects’ means to incorporate information into the model out the repeated measures (random effect). The resulting Z (Gaussianised T) statistic images were thresholded using an initial cluster-forming threshold of Z>3.1 (corrected cluster extent threshold of P<0.05).

For direct comparison of treatment groups we used measurements 2 and 5 weeks after the BCCAo.

Measurements acquired two weeks and five weeks after the BCCAo were compared with a general linear model, implementing a repeated measures ANOVA design with one fixed and one mixed effect. The effect-of-interest (fixed factor) was the (treatment donepezil) and additional covariates modeled the individual subjects’ means to incorporate information into the model out the repeated measures (random effect). The resulting Z (Gaussianised T) statistic images were thresholded using an initial cluster-forming threshold of Z>2.3.

### Analysis of diffusion tensor imaging (DTI) data

The DTI images were corrected for eddy current artifacts by FSL eddy_correct and were masked based on the brain-extracted, high-resolution anatomical image. The diffusion tensor model was fitted at each voxel within the mask by using FSL Dtifit. The resulting fractional anisotropy (FA) and mean diffusivity (MD) maps were spatially standardized. The voxel-wise statistical analysis of the FA data was carried out using TBSS (Tract-Based Spatial Statistics, [51]), which is part of FSL [52]. TBSS project all the subjects' FA data onto a mean FA tract skeleton before applying voxel-wise cross-subject statistics. To acquire the DTI voxel data from the white matter, the population-mean FA map was thresholded at 0.23, and the individual local maxima were projected onto the skeleton as described in [51]. The diffusion data in the skeleton were subjected to voxel-wise permutation test-based statistics (n = 10000) as implemented in FSL [47]. The statistical thresholding was carried out with threshold free cluster enhancement (TFCE) [53] and GRF-theory-based maximum height thresholding with a (corrected) significance threshold of P = 0.05 [50]. We calculated mean values based on the skeletonised FA an MD maps in the significant voxels, respectively.

### Voxel-based morphometry

The structural images were segmented into tissue types by FSL Fast [54]. For the voxel-wise atrophy analysis, we used an optimized VBM-style protocol [55, 56] in FSL [40]. Non-brain parts were removed from all the structural images [40], and the tissue-type segmentation was carried out by FAST4 [54]. To obtain optimal tissue classification, an in-house *a priori* tissue map was used. The volume of the whole-brain gray and white matter was estimated by taking the sum of the corresponding partial-volume estimate maps.

The resulting gray-matter partial volume images were registered to a standard space using linear transformation [39], followed by a non-linear registration [57]. The resulting images were averaged to create a study-specific template to which the native gray matter images were then non-linearly reregistered. The registered partial volume images were then modulated (to correct for local expansion or contraction) by dividing by the Jacobian of the warp field. The modulated, segmented images were then smoothed with an isotropic Gaussian kernel with a sigma of 4 mm. Finally, a voxel-wise GLM was used with permutation-based non-parametric testing. The thresholding was carried out by the TFCE technique [53]. The images were thresholded with GRF-theory-based maximum height thresholding at a (corrected) significance threshold of P = 0.05 [50]. We also calculated the average effect size based on the proton density images weighted by jacobian determinant in the significant voxels.

## Results

### Mortality and ischemic lesions

Two out of the 30 animals died 4 and 6 days after occlusion of the second carotid artery. Because this happened prior to the donepezil treatment, we could not differentiate the mortality between the treatment groups. Additionally, we did not find any difference in the weight of the animals after donepezil treatment (weight gain was found to be 51±14.7 g and 59±13.9 g in the donepezil and saline treated groups, respectively). The multimodal MR imaging revealed only one ischemic lesion in the left hippocampus in the case of one animal (in all measurements 1–5 weeks after the second carotid artery occlusion, saline group). We excluded this animal from the whole evaluation process because of extended ischemic lesion. Only this animal was excluded. Our exclusion criteria were: 1. anatomical abnormalities (i.e. larger or asymmetrical anatomical structures) 2. Extensive lesions, or any sign of extended tissue impairment) 3. We excluded animals from the evaluation process of MRI measurements if their movements during the fMRI measurement exceeded 2 mm.

### Gray matter atrophy

**Fig 2:**
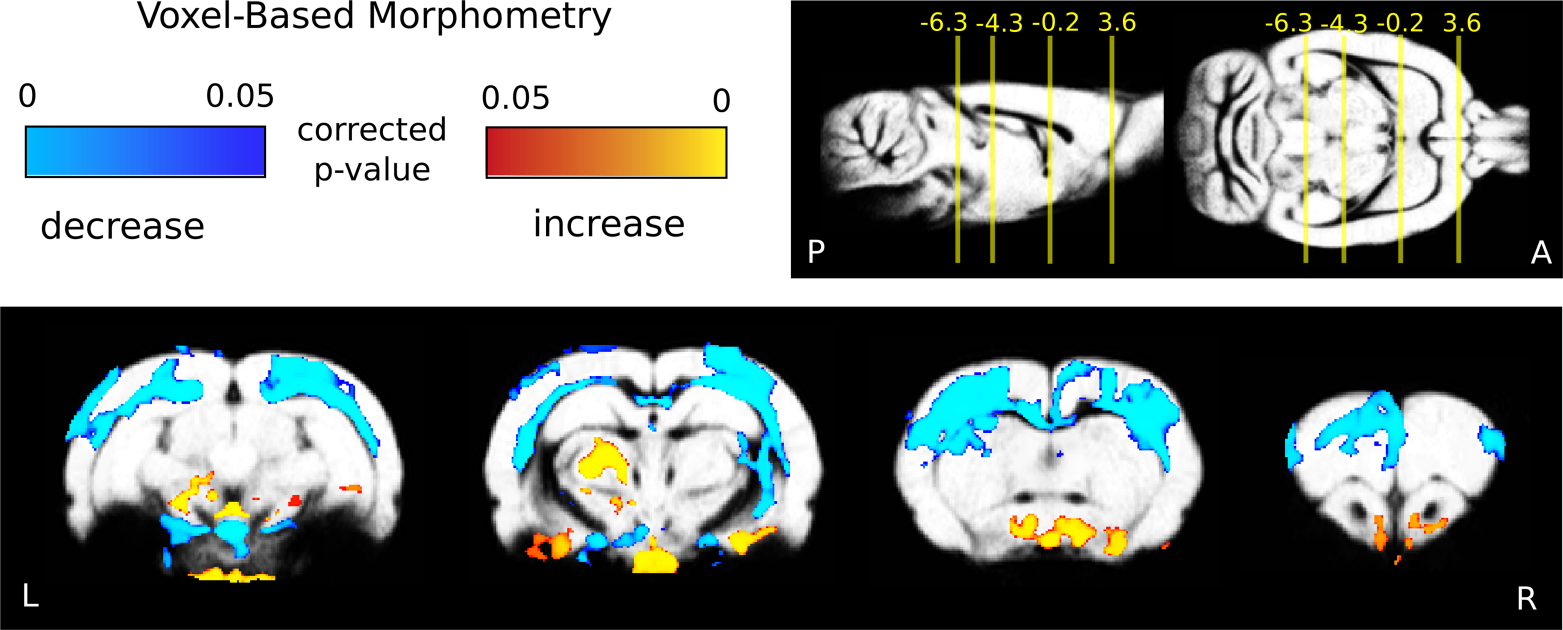
Gray matter atrophy 5 weeks after stepwise BCCAo. Blue-to-light-blue and red-to-yellow colors represent decreased and increased gray matter density 5 weeks after the BCCAo compared to baseline measurements, respectively (the treatment groups were pooled: n = 27 and n = 26, voxel-wise permutation test). Color bars represent p-values. The coordinates of the slices are represented in relation to bregma. The statistical images were enhanced using TFCE, corrected for multiple comparisons and overlaid on the population-mean gray matter probability map.

The VBM analysis revealed significant changes in several cortical and subcortical areas (Fig 2 and Table 1) five weeks after the BCCAo.

**Table 1:** VBM clusters and local maxima.

#	Size (mm3)	Xpeak	Ypeak	Zpeak	P	regions
**decrease**						
**1**	249	5.2	-5.5	-3.2	<0.001	Bilateral parietal and occipital lobes, striatum
**2**	16	-0.1	-6-7	-8.9	<0.001	Frontal pole
**Increase**						
**1**	141	0.4	-10.3	-10.1	<0.001	Thalamus, subthalamus, tegmentum, medulla, hypothalamus, right ventral hippocampus

In the first two columns of the table, the index and size of each VBM cluster is listed. The coordinates, P-values and overlapping anatomical regions are listed in 3–7. The clusters were formed on the TFCE images after multiple comparisons correction with a cluster-forming threshold of p<0.05. Only the clusters with a size greater than 1 mm are listed. The region names and peak coordinates are given following the orientation to the rat brain atlas of Paxinos and Watson. The treatment groups were pooled, baseline n = 27; 5 weeks after BCCAO n = 26.

An extensively decreased gray matter density (including areas of the frontal, parietal and occipital cortices and dorsal striatal areas) was observed (p<0.05, voxel-wise, TFCE enhanced, the treatment groups were pooled: n = 27 and n = 26, 1 rat was excluded because of movements,). The average effect size was (mean±sd): -8±2.6%. Increased gray matter volume in subcortical (ventro-postero-medial thalamic nucleus, posterior thalamic nuclear group), ventral brain areas was also detected (Fig 2 and Table 1).

There were no significant differences in the levels of gray matter atrophy between the two treatment groups 5 weeks after BCCAo (saline vs donepezil n = 13 and 13, 1 rat were excluded because of movement, total brain and voxel-wise analysis).

### White matter microstructural alterations

No significant differences in the diffusion parameter alterations were found between the treatment groups (donepezil vs saline, two weeks after BCCAo: n = 10 and 12, 3 and 2 rats were excluded because of movement; 5 weeks after BCCAo: n = 11 and 12, 2 and 2 rats were excluded because of movement) so we pooled the data for further analysis (baseline *vs*. BCCAo).

**Fig 3:**
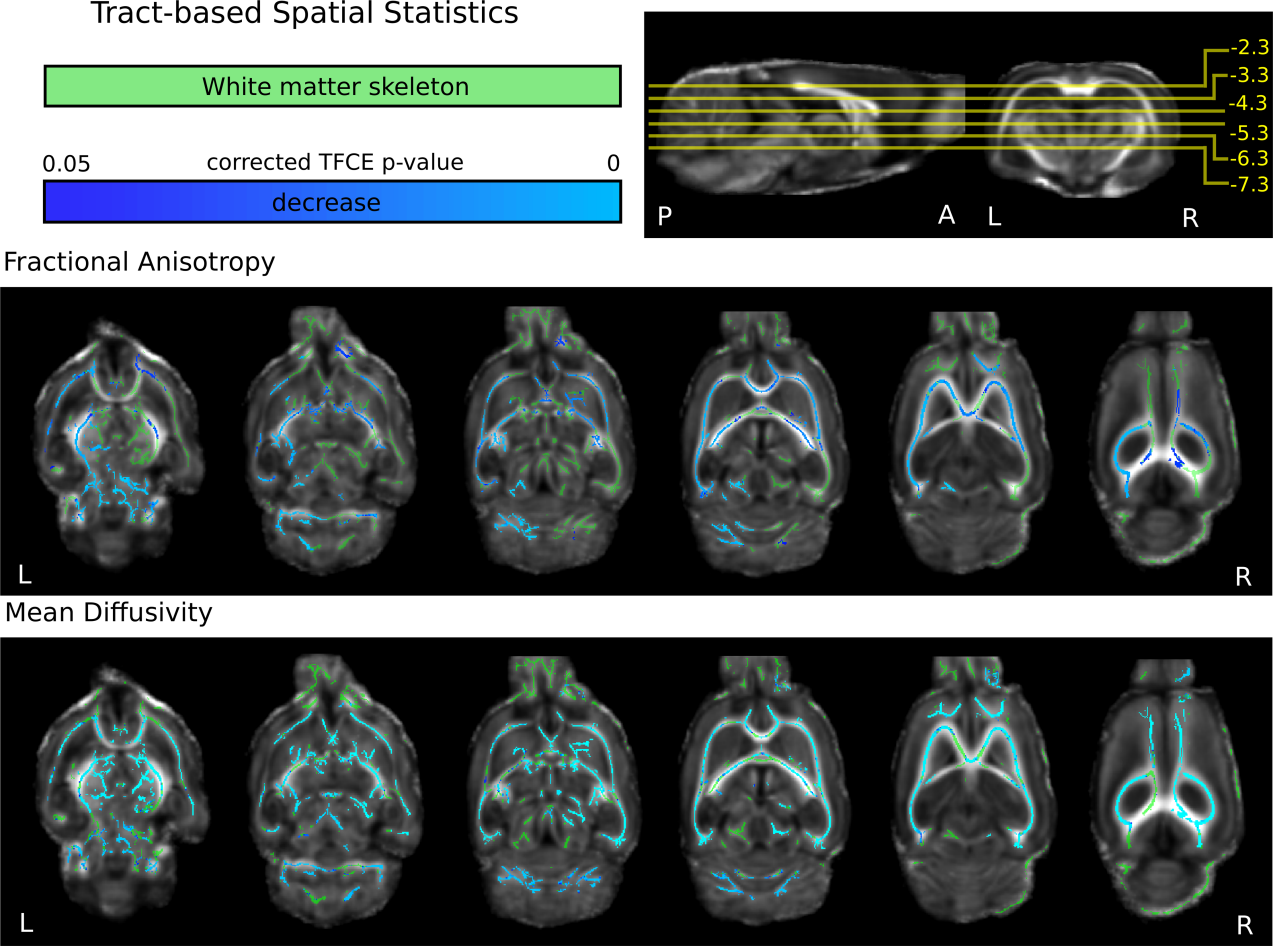
White matter microstructural changes 5 weeks after the stepwise BCCAo. Blue-to-light-blue colors represent reduced fractional anisotropy (FA, middle) or mean diffusivity (MD, bottom) over the white matter TBSS skeleton (green) 5 weeks after the BCCAo (n = 25), compared to the baseline measurements (n = 23, the treatment groups were pooled) The color bar represents p-values. The coordinates of slices are represented in relation to bregma. The statistical images are overlaid on the population-mean FA map.

Five weeks after the BCCAo, using TBSS (Tract-Based Spatial Statistics) we found significant changes in the average MD (mean diffusivity, Fig 3) and widespread alterations in the FA (fractional anisotropy, Fig 3) of the white matter (p<0.05, voxel-wise, TFCE enhanced, n = 25 vs. n = 23, the treatment groups were pooled, 2 and 4 rats were excluded because of movement,). We found significantly reduced FA and MD in various important neural tracts, including the optic tract, capsula interna, corpus callosum, septum, medial lemniscus and the medial forebrain bundle (S1 Fig). Average change of FA and MD values were—5.5±4.7%, and -6.6±5.5%, respectively.

We did not find any significant difference in structural white matter alterations two weeks after BCCAo (baseline: n = 21, 6 rats were excluded because of movement, and two weeks after BCCAo: n = 22, 5 rats were excluded because of movement).

### Effect of acute donepezil treatment on brain perfusion parameters as measured by DSC-MRI

**Fig 4:**
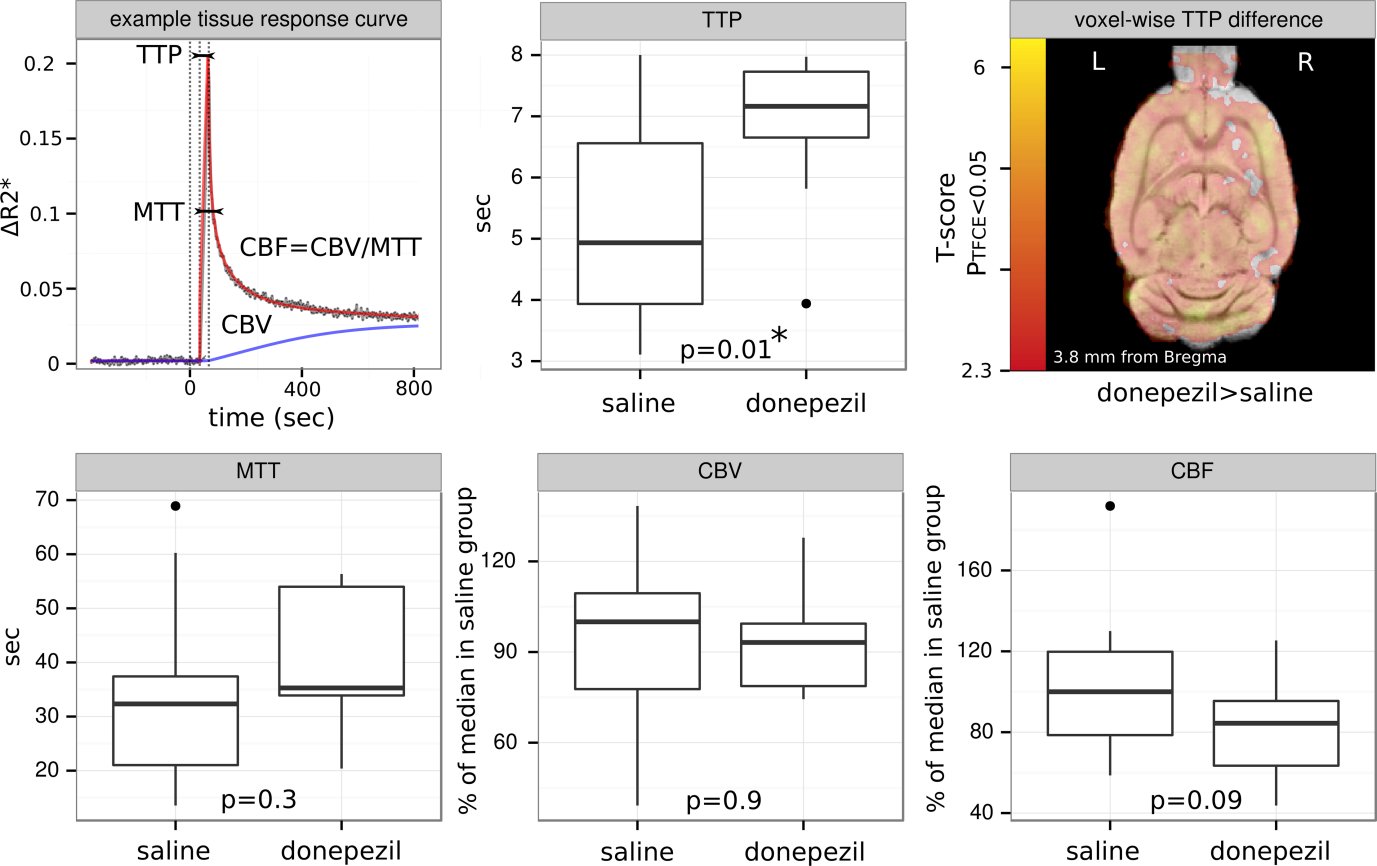
Effect of acute donepezil on whole brain perfusion parameters one week after the stepwise BCCAo. *Top*, *left*: Example gadolinium concentration-related ΔR2* tissue-response curve. The modeling of the measured ΔR2* as a linear mixture of a sigmoid basis for recirculation and a gamma-variate function for tissue-response allows the estimation of the time to peak (TTP), mean transit time (MTT), regional cerebral blood volume and flow (CBV and CBF), respectively. *Top*, *middle and right*: Whole-brain and voxel-wise differences in the TTP donepezil compared to saline treatment. Bottom: Whole-brain differences in the MTT, CBV and CBF, respectively. A significant (p = 0.01, saline: n = 13, donepezil: n = 12) difference was observed in the TTP between the saline-treated and donepezil-treated conditions, with the latter having a longer TTP.

We compared the DSC perfusion parameters between the donepezil- and saline-treated groups at two time points: after the first injection of donepezil (or saline, acute treatment—one week after the BCCAo, Fig 4) and 3 weeks later (2 weeks after the semi-chronic donepezil (3 mg/kg/day/8 days) or saline treatment–at 4 weeks after the BCCAo, see also Fig 1). At the first measurement, we found an increase in the time to peak (TTP, p = 0.01, saline: n = 13, donepezil: n = 12, 1 and 3 rats were excluded because of movement) of the whole brain after acute donepezil treatment. The level of isoflurane did not vary between the treatment groups (1.7±0.29% for saline and 1.7±0.26% for donepezil group). In our second DSC measurement 4 weeks after the BCCAo, the perfusion parameters did not significantly differ between the saline- (n = 11, 2 rats were excluded because of movement) and donepezil-treated (n = 13, 1 rats were excluded because of movement) groups.

### Changes in the BOLD responses to whisker stimulation in the two treatment groups

#### Saline group

**Fig 5:**
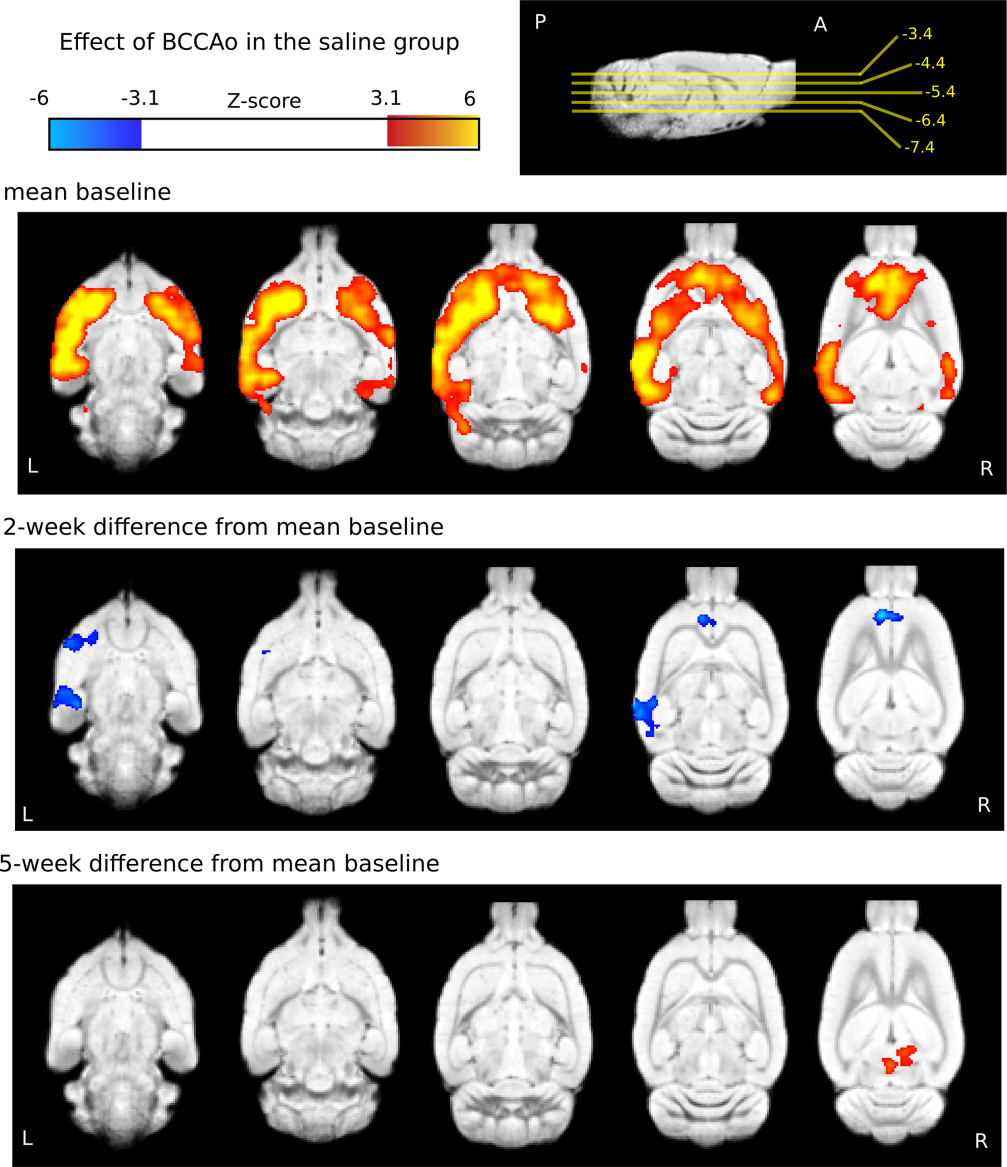
Mean baseline BOLD responses to whisker stimulation and their changes at 2 and 5 weeks after the stepwise BCCAo in the saline-treated group. The mean activation evoked by the sensory stimulation in the saline-treated group before the stepwise carotid occlusion (upper row, n = 12). Contrast images show decreased BOLD response 2 weeks after (2 weeks difference from mean baseline; middle row, n = 12) and restored BOLD response at 5 weeks (5 weeks difference from mean baseline; bottom row, n = 12) after the stepwise carotid occlusion. The statistical z-score images were thresholded at Z>3.1 to construct the activation clusters. Afterwards, a cluster extent probability threshold of p<0.05 was applied to correct for multiple comparisons. Blue-to-light-blue colors represent decreased, while red-to-yellow colors represent increased, BOLD responses. Color bars represent z-values. The coordinates of the horizontal slices are presented next to the color bar on a sagittal view in relation to bregma. The statistical images were overlaid on an in-house standard template [58].

Two large clusters survived the significance testing in the baseline condition of the animals later treated with saline. These clusters were found in areas involved in the somatosensory circuit, as well as in other cortical and subcortical regions (mean baseline, Fig 5, upper row, n = 12, 1 rat was excluded because of movement).

Two weeks after the BCCAo four clusters of decreased activity were observed and were localized in the striatum and in the prelimbic, piriform and auditory cortices in the saline-treated group (Fig 5, middle row, n = 12, 1 rat was excluded because of movement).

Five weeks after the BCCAo, increased activation was found in the retrosplenial granular cortex and in the cerebellum (n = 12, 1 rat was excluded because of movement). All results were thresholded at p<0.05 after correcting for multiple comparisons via cluster significance (TFCE) with a cluster-forming threshold of Z = 3.1; (Fig 5, Table 2).

**Table 2:** Local maxima of the mean BOLD responses to whisker stimulation in the saline group and their changes two and five weeks after the BCCAo.

#	Size (mm3)	Pclust	Xpeak	Ypeak	Zpeak	% signal Change	Z-score	region
**mean baseline**								
1	408	<0.0001	-3.3	0	-7.4	0.23	7.67	L striatum
			-6.9	-5.6	-4.4	0.31	7.66	L prim. auditory ctx.
			-5.5	-2.2	-7.2	0.2	7.12	R amygdala
			-5.3	-1.8	-7.2	0.2	7.02	L claustrum
			-2.5	0.6	-5.8	0.23	7.03	L striatum
			-6.5	-5	-7.4	0.18	7	L piriform ctx.
2	3,6	0,0471	1.9	-9.4	-2.2	0.27	5.21	R cerebellum
			2.7	-9	-2.4	0.21	4.74	R retrosplenial dysgranular ctx.
**2-week difference**								
1	9	0,00134	-2.7	0.8	-8.4	-0.16	-4.28	L striatum
2	7,7	0,00284	-0.9	3.4	-3.2	-0.29	-4.78	L prelimbic ctx.
3	6,2	0,00743	-6.7	-4	-8.2	-0.16	-4.47	L piriform ctx.
1	4,1	0,0321	-7.5	-5	-4.4	-0.18	-5.17	L prim. auditory ctx.
**5-week difference**								
1	9,3	0,00112	1.7	-7.2	-1.2	0.27	5.15	R retrosplenial granular ctx.
2	3,9	0,037	-0.1	-7.8	-3.2	0.16	4.08	L cerebellum

In the first three columns of the table, the index and size of the cluster and the corresponding cluster occurrence probability is listed. The coordinates, % signal change values and z-scores of the local maxima within the cluster are listed in columns 4–8. Column 9 denotes the anatomical location of the local activation maxima (according to the atlas of Paxinos and Watson). Only local maxima with z>4 are listed. When local maxima are closer to each other than 2 mm, only the one with the greater z-score is listed. All results were thresholded at p<0.05 after correcting for multiple comparisons via cluster significance with a cluster-forming threshold of z = 3.1 (n = 12).

### Donepezil group

**Fig 6:**
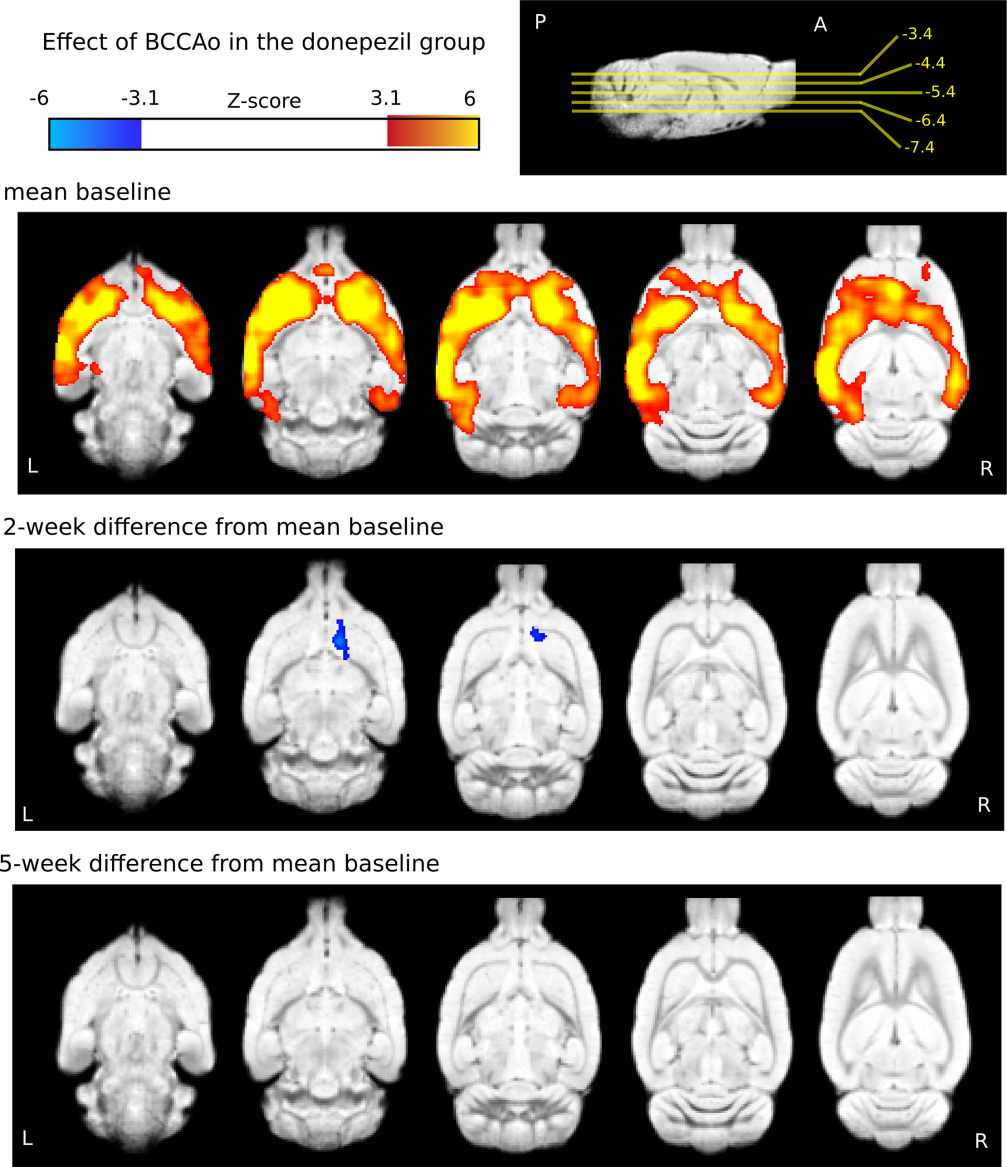
Mean baseline BOLD responses to whisker stimulation and their changes at 2 and 5 weeks after the stepwise BCCAo in the donepezil-treated group. The mean activation evoked by the whisker sensory stimulation in the donepezil-treated group before the stepwise carotid occlusion (upper row, n = 14). Contrast images show that the treatment ameliorated the drop in BOLD responses 2 weeks (middle row, n = 11) after and restored responses 5 weeks (bottom row, n = 13) after the stepwise carotid occlusion. The statistical z-score images were thresholded at z>3.1 to construct the activation clusters. Afterwards, a cluster extent probability threshold of p<0.05 was applied to correct for multiple comparisons. Blue-to-light-blue colors represent decreased, while red-to-yellow colors stand for increased BOLD responses. The color bars represent z-values. The coordinates of the horizontal slices are presented next to the color bar on a sagittal view in relation to bregma. The statistical images were overlaid on an in-house standard template [58].

In the animals later treated with donepezil, one large cluster survived the significance testing in the baseline condition (n = 14, Fig 6, upper row, mean baseline). The localization of the activation maxima was similar to that of the saline group (Fig 5 upper row, as expected, since none of the groups received treatment in the baseline condition; Fig 6, Table 3). In contrast, the two-week and five-week differences were not identical to those of the saline group. Two weeks after the BCCAo (at the end of the chronic donepezil treatment), the decrease in activation was not as widespread as in the saline group and was localized in the striatal areas as opposed to the cortical hypoactivations in the saline-treated group (n = 11, 3 rats were excluded because of movement). No significant differences were found five weeks after the BCCAo compared to the baseline activation in this group (Fig 6, Table 3, n = 13, 1 rat was excluded because of movement).

**Table 3:** Local maxima of the mean BOLD responses to whisker stimulation in the donepezil-treated group and their changes two and five weeks after the BCCAo.

#	Size (mm3)	Pclust	Xpeak	Ypeak	Zpeak	% signal Change	z-score	region
**mean baseline**								
**1**	509	<0.0001	2.9	0.4	-6.2	0.25	8.96	R striatum
			-4.7	-0.2	-6.2	0.24	8.92	L striatum
			-7.3	-5	-5.2	0.29	8.91	L secondary auditory ctx.
			-4.7	-0.4	-6.8	0.23	8.89	L striatum
			-5.1	0	-6.8	0.23	8.84	L claustrum
			-3.9	0	-6	0.25	8.82	L striatum
**2-week difference**								
**1**	4.3	0.033	1.3	0.4	-6.8	-0.1	-4.21	R striatum
**5-week difference**								
**-**	-	-	-	-	-	-	-	-

All results were thresholded at p<0.05 after correcting for multiple comparisons via cluster significance with a cluster-forming threshold of z = 3.1. (baseline: n = 14, two weeks after BCCAo: n = 11, five weeks after BCCAo: n = 13). In the first three columns, the number and size of the cluster and the corresponding cluster occurrence probability are listed. The coordinates, % signal change values and z-scores of the local maxima within the cluster are listed in columns 4–8. Column 9 denotes the anatomical location of the local activation maxima. Only the local maxima with z>4 are listed. When the local maxima were closer to each other than 2 mm, only the one with the greater z-score is listed. The region names and peak coordinates are given following the orientation of the rat brain atlas of Paxinos and Watson [59]

### Direct comparison of the evoked BOLD responses between the two groups–the effects of semi-chronic donepezil treatment

**Fig 7:**
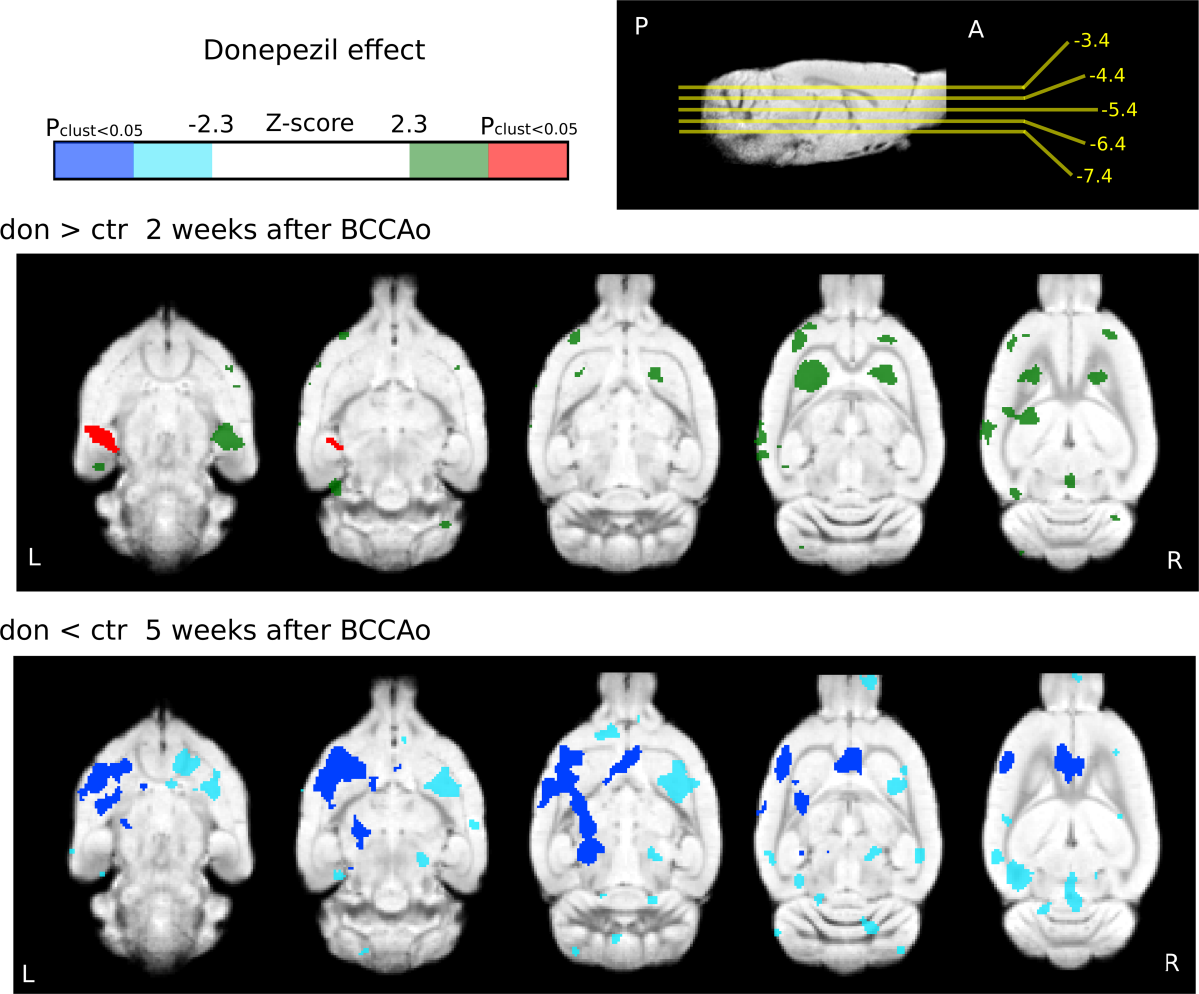
Direct comparison of the BOLD responses to whisker stimulation between the donepezil- and saline-treated groups. The statistical z-score images were thresholded at z>2.3 to construct the activation clusters. These (uncorrected) clusters are displayed in green (increased activation) and light blue (decreased activation). Then, a cluster extent probability threshold of p<0.05 was applied to correct for multiple comparisons. Dark blue represents significant (corrected) clusters of decreased activation in the donepezil group, whereas red colors stand for significant (corrected) clusters of increased BOLD responses (saline: n = 12, donepezil: n = 13). The coordinates of the horizontal slices are presented next to the color bar on a sagittal view in relation to bregma. The statistical images were overlaid on an in-house standard template [58].

As an outcome of daily administration of donepezil (3 mg/kg i.p.) for 8 days, one significant cluster of increased activity in the donepezil-treated group was observed two weeks after the BCCAo, compared to the saline-treated group. This change in activation can be regarded as an improvement in the decline of the somatosensory BOLD response in the ventral hippocampal areas, as seen in the saline group in response to the whisker stimulation 2 weeks after the occlusion (saline: n = 12, donepezil: n = 11; Figs 5–7, Tables 2–4).

**Table 4:** Local maxima of the BOLD response differences between the donepezil- and saline-treated groups two and five weeks after the BCCAo.

Cluster	Size (mm3)	Pclust	Xpeak	Ypeak	Zpeak	% signal Change	z-score	region
**don > ctr 2 week**								
**1**	15	0.0471	-3.7	-0.4	-9.8	0.1	3.75	L hippocampus
**don < ctr 5 weeks**								
**1**	58.4	<0.0001	0.2	1	-4	-0.05	4.23	anterior cingulate ctx.
**2**	21.8	0.012	-4.1	-0.6	-5.6	0.01	4.25	L insular ctx.

All the results were thresholded at p<0.05 after correcting for multiple comparisons via cluster significance with a cluster-forming threshold of z = 2.3. In the first three columns, the number and size of the cluster and the corresponding cluster occurrence probability are listed. The coordinates, % signal change values and z-scores of the local maxima within the cluster are listed in columns 4–8. Column 9 denotes the anatomical location of the local activation maxima. Only the local maxima with z>4 are listed. When local maxima were closer to each other than 2 mm, only the one with the greater z-score is listed. The region names and peak coordinates are given following the orientation of the rat brain atlas of Paxinos and Watson[59].

Five weeks after the BCCAo, an activation decrease was observed in the donepezil group compared to the saline group (saline: n = 12, donepezil: n = 13; Fig 7, Table 4). Let us note that when assessing the effect of donepezil, we used a lower cluster-defining threshold (z = 2.3) in order to be able to detect these small magnitude changes. Thus, in this statistical contrast, there is a greater chance of false positives. However, the pattern of bilateral differences in the ventral hippocampus (Fig 7) suggests that the observed activation changes, or at least those localized in the hippocampus (two weeks after the BCCAo), are true positive changes.

### Effect of stepwise BCCAO on visual evoked potential

In a supplementary study (n = 8, BCCAo and n = 5 sham) we observed the drastic disappearance of visually evoked responses measured over visual cortical areas (S2 Fig, S1 Text).

## Discussion

In this multimodal MRI study, we longitudinally monitored the effects of chronic hypoperfusion in the rat stepwise BCCAo model. The chronic hypoperfusion induced significant white matter microstructural alterations and gray matter atrophy revealed by TBSS analysis of DTI data and VBM analysis of high-resolution anatomical images. In this model, we found a decreased BOLD response to whisker stimulation at 2 weeks after the occlusions in various cortical, striatal and thalamic brain areas, which were restored by the 5th week. The semi-chronic administration of donepezil reversed the decline in the BOLD fMRI response in the ventral hippocampal areas measured immediately after the last dose of donepezil administration at 2 weeks after the occlusion, but did not modify the structural alterations of the grey and white matter.

In a similar longitudinal multimodal MRI study of the classical BCCAo model, Soria and coworkers emphasized the frequent (70% of the rats) occurrence of large ischemic lesions in the striatum [26] while other studies reported massive hippocampal lesions [11]. It is also well-known that the mortality rate is high in the classical, instantaneous BCCAo model [11, 19] and to circumvent this, the stepwise occlusion has been introduced, which indeed reduced the mortality rate to 8% [29–33] that strictly resembles the small mortality rate measured in this study (7%). Notably, our detailed anatomical scans 2 and 5 weeks after stepwise BCCAo did not reveal large, extended ischemic lesions observed previously after classical BCCAo [11, 26].

Due to the compensatory mechanisms activated after the first occlusion, the stepwise occlusion is assumed to promote a more gradual decrease in brain perfusion [27]. Indeed, in the classical instantaneous BCCAo model, various studies have shown a sudden decrease in CBF that is relatively severe during the first 1–3 days [11, 13, 28]. Importantly, in the classical model, the cognitive dysfunction develops around the second week and progresses for several weeks, despite restoration of the CBF [11, 12, 16, 17, 19, 60, 61]. In the stepwise occlusion model, the CBF returns to the preocclusion level around the third week in the cortex, striatum and cerebellum, as measured with 3D ASL (arterial spin labelling), which is in harmony with the gradual enlargement of the diameter of the vertebral artery, as measured with time-of-flight (TOF) MR angiography [27]. So acute reduction of cerebral blood flow was found to be slightly smaller (about 50% *vs* 60%,) and the compensation of cerebral perfusion was faster (3 weeks *vs* 6–8 weeks) after the stepwise occlusion, supporting that it could really initiated more gradual and less serious acute hypoperfusion [27]. We suppose that some biochemical mediators might have been included, but further experiments are needed to answer this question.

Alongside previously demonstrated long term cognitive problems [30–33] there is a further progressive decline in the number of microvessels and additional synaptic proteome changes in the cortex detected 7 weeks after the stepwise BCCAo [27, 62]. These data suggest, that the stepwise model, while providing less acute effects (e.g., stroke or mortality), induces relevant biochemical changes, e.g. altered synaptic apolipoprotein E (APOE) relevant to the human hypoperfusion.

Donepezil, an acetylcholinesterase inhibitor, could successfully improve the cognitive performance of hypoperfused rats [16, 17]. In previous human studies, donepezil effectively improved both CBF and cognitive performance in chronic treatment models [63, 64]. In contrast, in our DSC-MRI measurements, a single dose of donepezil increased the time-to peak (TTP) thus worsened the cerebral blood flow at least transiently. Donepezil might achieve this by influencing the cerebral perfusion via cholinergic modulation of the cerebral vasculature as arteriolar dilation by donepezil has been presented *in vitro* [65].

To minimize the side effects of the repeated long-term anesthesia and the repeated i.v. injection of contrast agent (Gadovist, DSC-MRI) we needed to optimize our measurements. Thus we measured brain perfusion only at two time points: after the first donepezil injection (acute effect) and two weeks after the semi-chronic treatment, to reveal the possible long term effects of donepezil on the model.

Although one single dose of donepezil has been shown to be effective in object recognition memory test and modulating EEG activity in rats [66, 67], in humans chronic administration is used. In rats the semi-chronic administration of donepezil (3mg/kg 8 days) has been demonstrated to improve memory after stepwise occlusion, [16] therefore we addressed this treatment mode. Primarily we evaluated its effect on the whisker stimulation-evoked BOLD fMRI response. Donepezil efficiently improved the transient reduction of the evoked BOLD response that was measured immediately after the last dose of the semi-chronic donepezil administration, 2 weeks after the occlusion. While donepezil definitely influences the tone of the cerebral vasculature through its cholinergic effect, this effect of donepezil treatment could very well have a more direct neuronal effect since ventral hippocampal BOLD activation was found when the donepezil treatment was contrasted directly to the control saline treatment. Notably, the effect of donepezil on the BOLD response did not persisted after the cessation of its administration; in fact, it slightly decreased the BOLD response 5 weeks after the BCCAo in the donepezil-treated group (as compared to saline-treated group), suggesting that the disruption of the donepezil treatment might worsen the outcome as well.

Our whisker stimulation-evoked BOLD response peaked in the somatosensory cortex and covered various additional cortical and subcortical areas [58]. The BOLD signal reflects a complex interplay of neuronal and vascular components of activation; hence the reduced BOLD activation could be derived from impaired hemodynamic coupling, decreased vascular reactivity or reduced neuronal activation, or a combination of these. In our study, the BOLD responses to whisker stimulation revealed transiently compromised functional responses 2 weeks after the occlusion. Several studies have demonstrated reduced BOLD activation after stroke or in patients with carotid stenosis [68–71]. Consistently, in rats, after transient global ischemia, impaired temporal initiation and a smaller activation area of the sensory stimulation-evoked functional CBF response was demonstrated [72]. Nonetheless, even in the classical BCCAo model, the acute ischemic period persists only for the first few days [11]; thus, in our case of stepwise occlusion without focal ischemic lesions, the functional alteration is caused by the direct or indirect effect of the chronic hypoperfusion. At the end of our study, the BOLD response was restored completely, compensatory mechanisms could have repaired the measured sensory processing domain, or the underlining neurovascular coupling, or vascular reactivity. Nevertheless, this suggests that the deterioration of the measured sensory processing domain (stimulation of whiskers) would not be directly involved in the well-described progressive deterioration of cognitive performance in this model.

In human investigations, brain atrophy was proposed to be one of the key factors behind the cognitive dysfunction [73, 74]. Voxel-based morphometry (VBM) was designed to be sensitive to structural differences and became an established tool in humans and lately in rats [55, 75–78]. Changes of tissue density correlate structural differences among groups calculated after spatial standardization. We have assessed the chronic hypoperfusion-induced brain atrophy by measuring the structural alterations in the gray matter with VBM in the rat stepwise BCCAo model, 5 weeks after the stepwise occlusions. We have found significant gray matter atrophy in widespread cortical areas, including the frontal, parietal and occipital cortices, and alterations in the dorsal striatum. This later finding may suggest possible neuronal loss in the dorsal striatum, despite the lack of large ischemic lesions, which is in agreement with the histological findings in this stepwise BCCAo model [27]. Our VBM result in the cortex is in agreement with the recent proteomics finding of 46 altered protein abundance in the synaptosome fractions of the cerebral cortex 7 weeks after the stepwise BCCAo occlusion, due to the chronic hypoperfusion [62].

In the classical, instantaneous BCCAo model of chronic cerebral hypoperfusion, the irreversible disruption of the white matter and myelin degradation has been suggested to be responsible for cognitive dysfunction that is correlated with the myelin changes [11, 61]. In humans, while the carotid stenosis-related atrophy was lateralized to the side of the stenosis [79], the white matter disintegration, as measured by diffusion tensor imaging, was found more extensively [80]. Furthermore, the parallel dynamic changes in cognitive function and in the white matter diffusion parameters after endarterectomy propose white matter microstructural measures as a potential biomarker [81].

We have assessed the chronic hypoperfusion-induced white matter microstructural changes using DTI measurements and TBSS analysis 5 weeks after the stepwise BCCAo. We have found significant changes 5 weeks after BCCAo in the average MD (mean diffusivity) and widespread alterations in the FA (fractional anisotropy). These results are in agreement with the longitudinal DTI study of the classical BCCAo model, where the optic nerve, optic tract and external capsule white matter abnormalities were studied in detail [25]. However, our TBSS analysis was more sensitive than previously used voxel based comparisons [25, 26] and revealed extensive microstructural alterations throughout the whole white matter skeleton after the stepwise BCCAo. DTI measures water diffusion in tissues, which is highly sensitive to differences in the microstructural architecture of cellular membranes [82]. FA is a summary measure of microstructural integrity, so FA alterations sensitively reflects microstructural changes of the white matter, but doesn’t provide information about the type of the changes. MD, as an inverse measure of membrane density reflects cellularity, altered MD could be observed during edema or necrosis [82]. Note however, that the exact nature of the cellular changes could not be described without histological examinations.

The detailed mechanism of the white matter disintegration and the gray matter atrophy is not entirely known yet. In the classical BCCAo model, it was proposed that the vascular events activate angiotensin II [6], propagate neuroinflammation and microglial activation [83], producing reactive oxygen species [7, 84]. These processes eventually lead to neurodegeneration, oligodendrocyte degeneration and the destruction of the myelin sheath [84]. Smaller degree of neurodegeneration, astrogliosis and oxidative stress has been reported after stepwise BCCAO [30–32] suggesting that similar mechanisms could be involved in the grey and white matter changes in this model. It could be of particular importance that the progressive microstructural changes in the white matter, as was mentioned in connection with the classical BCCAo model, likewise included a severe optic tract degeneration, as has been revealed by multiple DTI and histological studies [25, 26, 85]. In our stepwise occlusion model, degradation of visual function has not been demonstrated yet, so we began a separate study to determine changes in visually evoked EEG responses after stepwise occlusion. In a supplementary study, we found a drastic disappearance of visually evoked responses measured over visual cortical areas in BCCAo rats (S2 Fig), revealing that the stepwise occlusion did not prevent the visual function deterioration of these rats. Therefore, caution should be exercised even with the stepwise BCCAo rats when testing them with behavioral tests, though their cognitive abilities can still be revealed by testing their navigation in the absence of visual cues in total darkness, for example [86].

In conclusion, the stepwise BCCAo model examined in this multimodal MRI study provided a chronic hypoperfusion model without acute ischemic complications (e.g., stroke or mortality) that better resembles the human chronic hypoperfusion that would contribute to the cognitive deterioration of the brain. Furthermore our fMRI study demonstrated a measurable effect of the semi-chronic donepezil treatment after stepwise BCCAo in rats, although it did not persisted and had no measurable influence on the morphological alterations. Nevertheless these results emphasize the translational value of multimodal MRI in the precise, objective evaluation of the model.

### Limitations

No behavioral experiments were made, thus the direct comparison of behavioral measures and the demonstrated structural and functional changes can not be done. No histological examinations were made, thus the cellular lever changes behind the observed structural alterations can not be disclosed.

Although changes in CBF has been extensively studied after BCCAo [11, 13, 28] and even after stepwise BCCAo [27] it would have been reasonable to monitor CBF alterations after BCCAo in our experiments as well. However in our design, the DSC-MRI measurements required the repeated use of the contrast agent Gadovist via the tail vein of the animals, and the third insertion of a hypodermic needle into the tail vein is not plausible. Furthermore, the planned measurements required long-lasting MRI scans, and to minimize the side effects of the repeated long-term anesthesia [87] we needed to optimize our measurements. Thus we measured brain perfusion only at two time points: after the first donepezil injection (acute effect) and two weeks after the semi-chronic treatment, to reveal the possible long term effects of donepezil on the model.

## Supporting information

S1 Fig Changes in fractional anisotropy and mean diffusivity in different white matter structures 5 weeks after stepwise BCCAo. Blue and green colors represent significantly reduced fractional anisotropy (FA, up) and mean diffusivity (MD, bottom) over the white matter TBSS skeleton 5 weeks after BCCAo as compared to baseline measurements. The coordinates of slices are represented in relation to the bregma. Statistical images overlaid on the population-mean FA map. Abbreviations: ec- external capsule, cc-corpus callosum, s-septum, st-striatum, aca–anterior commissure, fi-fimbria, OT–optic tract, ic–internal capsule, ml-medial lemniscus, mfb–medial forebrain bundle, cg—cingulum.
(TIF)

S2 Fig Visual evoked responses measured in the visual cortex (VEPs) after single 1 sec LED flash stimulation in sham operated and BCCAo rats. **A**: positions of the electrodes on the rat’s skull, **B**: representative averaged (400 trial) responses measured in sham operated (top) and BCCAo (bottom) rats. **C**: grand average of the VEP amplitudes measured at the minimum of 2 sessions during 2 days in each animal (mean ± sem). Gray area shows average noise amplitude.
(TIF)

S1 Text Methods (electrophysiology). (DOCX)
