# Supplementary figures and images for: Stepwise occlusion of the carotid arteries of the rat: MRI assessment of the effect of donepezil and hypoperfusion-induced brain atrophy and white matter microstructural changes
Source: PLoS One. 2018 May 31;13(5):e0198265. doi: 10.1371/journal.pone.0198265 (PMC5979036; doi:10.1371/journal.pone.0198265)

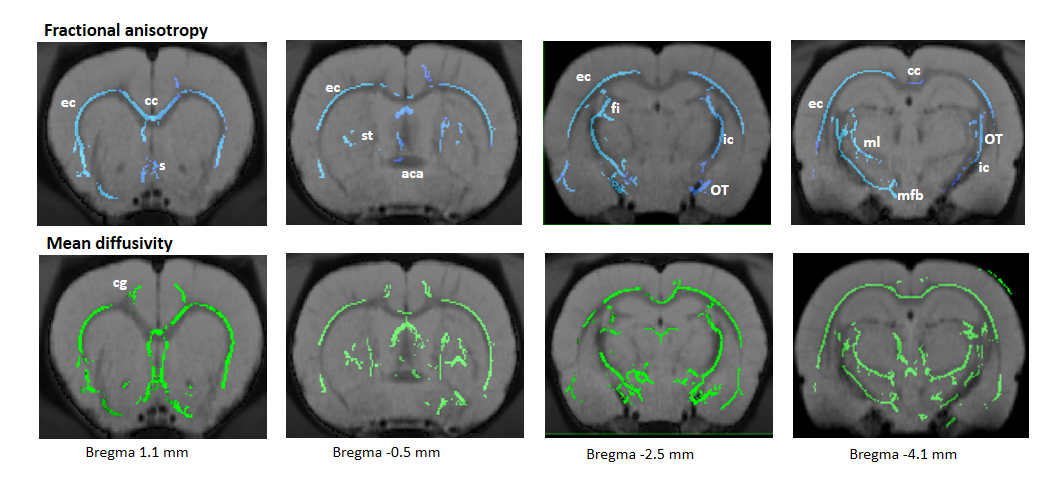

Supplement: S1 Fig — Blue and green colors represent significantly reduced fractional anisotropy (FA, up) and mean diffusivity (MD, bottom) over the white matter TBSS skeleton 5 weeks after BCCAo as compared to baseline measurements. The coordinates of slices are represented in relation to the bregma. Statistical images overlaid on the population-mean FA map. Abbreviations: ec- external capsule, cc-corpus callosum, s-septum, st-striatum, aca–anterior commissure, fi-fimbria, OT–optic tract, ic–internal capsule, ml-medial lemniscus, mfb–medial forebrain bundle, cg—cingulum. (TIF) [file pone.0198265.s001.tif]

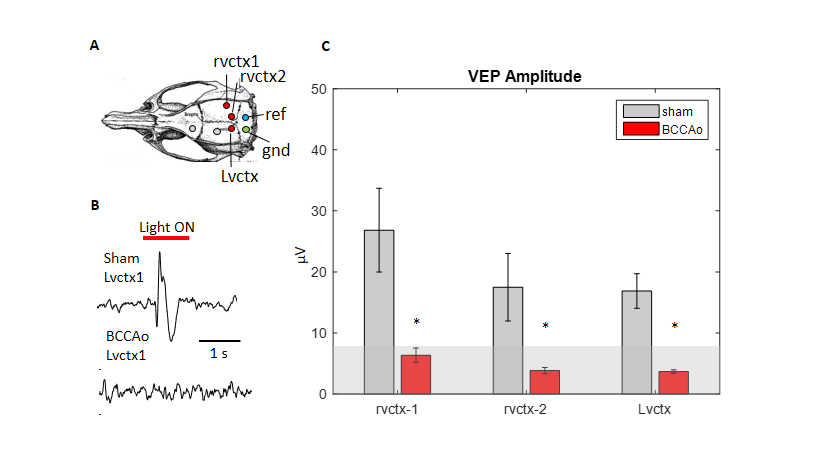

Supplement: S2 Fig — A: positions of the electrodes on the rat’s skull, B: representative averaged (400 trial) responses measured in sham operated (top) and BCCAo (bottom) rats. C: grand average of the VEP amplitudes measured at the minimum of 2 sessions during 2 days in each animal (mean ± sem). Gray area shows average noise amplitude. (TIF) [file pone.0198265.s002.tif]
