# Supplementary material for: Stepwise occlusion of the carotid arteries of the rat: MRI assessment of the effect of donepezil and hypoperfusion-induced brain atrophy and white matter microstructural changes
Source: PLoS One. 2018 May 31;13(5):e0198265. doi: 10.1371/journal.pone.0198265 (PMC5979036; doi:10.1371/journal.pone.0198265)
Supplement: S1 Text — (DOCX) [file pone.0198265.s003.docx]

**Supplementary Methods**

**Surgery**

In a separate study 12 (n=7 BCCAo and n= 5 sham operated) Wistar rats (weight 300-350 g) were used to measure visually evoked cortical responses. Stepwise 2 carotid occlusion were performed as described earlier. 6 month after the occlusion of the second carotid artery stainless steel screw electrodes (diameter 1.4 mm) were implanted above frontal (A: 1.5, L:2) parietal (A:-3, L:2) and visual (A:-6.5, L 1.5, A -5.5, L 5) cortex during isoflurane (1,5-2% in air) anesthesia. Reference and ground electrodes were placed above the cerebellum.

**Evoked Responses**

1 s long red LED light flashes were applied to dark-adopted rats in every 10 s as visual stimulation (the distance between the animal and LEDs was approximately 1 m). Data were recorded using Cambridge Electronic Device (CED) and Signal6 software (sampling frequency 5 kHz, filtered 0.16 Hz -1 KHz). One session included 400 trials, and we usually measured 2-3 session per day. Each animal was recorded through 2-3 days. Evoked responses were then averaged over sessions, and amplitudes of the responses were measured between the minimum value of the first negative peak to the maximum value of the first positive peak. Visually evoked potentials were then averaged and evaluated using built in functions in Signal, and further analysis were performed in Matlab. * p<0.05 measured by ANOVA.
